# NF-kappa B expression in resected specimen of colonic cancer is higher compared to its expression in inflammatory bowel diseases and polyps

**DOI:** 10.1038/s41598-022-21078-7

**Published:** 2022-10-05

**Authors:** Liron Berkovich, Mirit Gerber, Aviva Katzav, Debora Kidron, Shmuel Avital

**Affiliations:** 1 Department of Surgery B and Cancer Research Lab, Meir Medical Center, Kfar Saba, Israel; 2 The Sackler Faculty of Medicine, Tel Aviv University, Tel Aviv, Israel; 3 Department of Pathology, Meir Medical Center, Kfar Saba, Israel

**Keywords:** Cancer, Biomarkers, Gastroenterology, Medical research, Oncology, Pathogenesis

## Abstract

NF-Kappa B has a significant role in inflammatory processes as well as in colorectal cancer. The aim of this study was to compare the expression of NF-kappa B in colonic adenocarcinoma specimen, colonic adenomas and inflammatory colonic tissues. Patients with colorectal cancer (CRC), colonic adenomas and inflammatory processes undergoing surgery were recruited. Following a routine pathological evaluation tissue samples were stained using anti NF-κB monoclonal antibodies. Expression of NF-κB was quantified using IMAGEJ program for immunohistochemistry staining. Samples were also stained and quantified for CEA expression. Fifty-six patients were included. 30 cancers, 6 polyps and 20 inflammatory processes. Expression of NF-κB was similar between polypoid and inflammation etiologies. However, it was significantly higher in CRC compared to both (*p* < 0.05). In cancer patients, NF-κB expression in the resection margins was correlated with positive node status. CEA expression was higher in the cancer group, less in the IBD group and the lowest in the colonic non diseased margins. Our results provide a supportive evidence that NF-κB pathway is strongly involved in colon cancer development and metastasis. Interestingly, expression of NF-κB in benign polypoid lesions was as high as in inflammatory etiologies. This support the role of NF-κB early in the adenoma to carcinoma sequence. Further research is needed to evaluate the exact role of NF-κB in tumor progression in order to look for diagnostic and therapeutic possibilities.

## Introduction

The link between inflammation and tumorigenesis is well-established. It was first described in epidemiological studies indicating that chronic inflammation predisposes individuals to different cancers, including Colorectal Cancer (CRC)^1,2^. This was followed by evidence of how non-steroidal anti-inflammatory drugs (NSAIDs) decrease the prevalence of several tumors [reviewed in 1]. Finally, numerous mechanistic links between the two began to emerge, including NF-κB, which is discussed here^1,3^. This association has been demonstrated in several tissue types and organs, including the colon^1^.

CRC is one of the most common types of cancer in western countries. Sporadic, as well as colitis-associated CRC, develop gradually and present treatment challenges and greater likelihood of mortalityin advanced stages^4^. Much research has focused on characterizing new markers for early detection and staging of this type of cancer^2^. In parallel, in the context of high-risk IBD patients, a novel follow-up tool for early detection of the inflammation to cancer transition would have high clinical value.

NF-κB is an intracellular transcription factor with a distinct role in several processes related to inflammation and cancer^3^. NF-κB can become activated in response to extracellular stimuli, such as cytokines, growth factors, oncoproteins and stress signals^5^. Tumor necrosis factor alpha (TNFα) is the most well-known factor that triggers the NF-κB signaling pathway via its receptor. Other activating factors include interleukin-1 and toll-like receptor ligands.

Following activation, the NF-κB intracellular cascade can act both in the cytoplasm and in the nucleus to stimulate family proteins and target genes, thereby generating its diverse effects^6^.

In cellular models for colon cancer, induction of NF-κB was shown to promote tumor growth by upregulating and cross-talking with key signaling pathways, such as the phosphoinositide 3-kinase (PI3K)/Akt cascade, the cell-cycle process and anti-apoptotic pathways. NF-κB is also able to prompt production of proangiogenic and fundamental invasiveness factors, including cyclooxygenase-2 (COX2), growth factors, interleukins, cell adhesion molecules and matrix metalloproteinases^6,7^.

Involvement of NF-κB in all these pathways has a major effect on immune and inflammatory processes^8^. Namely, the NF-κB pathway is involved in the development of primary lymphoid organs and in the early formation and subsequent long-term maintenance of immune tolerance. It enables antigen-presenting cells of the innate immunity to communicate with the adaptive immunity, which is required for the survival and maturation of B cells in peripheral lymphoid organs (i.e., humoral immunity) and for regulating T cell responses (i.e., adaptive immunity).

Since NF-κB has a substantial role in inflammatory processes and in cancer development, this study aimed to evaluate NF-κB expression in colonic tissue of patients with colorectal cancer, IBD and colonic polyps. A comparison of NF-κB expression between the three enteritis was performed by quantifying the immunohistochemically staining.

In parallel we have looked also on CEA expression in these tissues as CEA is known to be overexpressed also in both IBD^9^ and in colorectal cancer^10^.

## Methods

This prospective, non-interventional study was approved by the Meir Medical Center Institutional Review Board, and was performed in accordance with the ethical standards laid down in the Declaration of Helsinki. All patients provided written informed consent before being included in this study.

Patients with malignant or benign colorectal diseases who were referred for elective surgery at Meir Medical Center from 2012 to 2018 were consecutively recruited to enroll in this non-interventional cohort study. AJCC staging system was used to characterize CRC patients^11^. Patients with distant metastasis discovered before or during surgery and those with positive macroscopic or microscopic resection margins were excluded.

### Immunohistochemically staining for NF-κB

The colorectal resection specimens were submitted for histopathological examination, according to routine protocol. All tissue samples were reviewed for each case. Two paraffin blocks were selected for the project: (a) pathological tissue from the primary tumor or the site of the benign pathology and (b) and from surgical margins. Four 4 µm-thick sections were cut from selected tissue blocks, embedded in xylene and dehydrated. They were further processed for NF-κB immunohistochemistry staining, according to the manufacturer’s protocol (MERCK Anti-NF-κB, P65, clone E379. Monoclonal Antibody. Millipore™) with an antibody dilution of 1:50,000. The immunostains were carried out on using Ventana BenchMark Ultra Automated Stainer (Roche Diagnostics, Basel, Switzerland).

As a positive control for NFkB staining we used mammary lymph nodes and for negative control primary antibodies were excluded from the staining procedure. all in accordance with the manufacturer's instructions and the supervision of our Pathology immunohistochemistry specialist.

### Quantification of tissue NF-κB levels

Digital images were captured from the stained slides using the Olympus BX41 microscope, equipped with an Olympus DP73 camera and were 4 times magnified . Captures of four different fields were taken from every slide (× 4, 381*513 sq-pixels) and saved using Olympus Entry cellSens software^12^. NF-κB tissue quantification levels were calculated using IMAGEJ program for IHC, using the ImageJ Colour Deconvolution filter to distinguish NF-κB staining from hematoxylin and separate DAB staining^13,14^. Data values were normalized per area and then converted to optical density (OD) measurements using the Rodbar function^15^:

$${\text{OD}} = \log {\text{ }}({\text{max}}\,{\text{intensity}}/{\text{mean}}\,{\text{intensity}}),{\text{ where}}\,{\text{max}}\,{\text{intensity }} = {\text{ }}255\,{\text{for}}\,8 - {\text{bit}}\,{\text{images}}.$$

Captured fields and/or missing tissue may deviate and cofound calculations. Thus, we selected only fields containing tissue either with no empty areas or those as small as possible.

### Statistics

Statistical analysis was performed using Statistical Package for Social Sciences, Version 25.0 (IBM, Armonk, NY, USA). The data are described as numbers and percentages for nominal parameters and as means and standard deviations for continuous variables.

Differences between characteristic qualitative variables and NF-κB levels were compared using t-test and Mann–Whitney U test. Mean optical density, standard deviation and p-values in Mann–Whitney correlations are shown in the tables and figures. Correlations between two continuous variables were evaluated with Pearson's correlation and Mann–Whitney test. Chi-square test was used to analyze categorical variables. P-values less than 0.05 were considered statistically significant.

## Results

A total of 56 patients were included in this study, 30 with a CRC diagnosis and 26 with benign conditions of the colon (Table 1).

**Table 1:** Patient characteristics.

Characteristic	CRC	Benign
Total, n	30	26
Age, years, (mean ± SD)	70 ± 12	48 ± 18
Sex (male, female)	16, 14	12, 14
BMI (mean ± SD)	28.0 ± 4.4	22.4 ± 5.5
Smoking, n (%)	5 (16%)	12 (46%)

**Etiology** (n)	Adenocarcinoma	Benign
	Stage I, 6 (20%)	IBD, 16 (58%)
	Stage II, 15 (50%)	Polyp, 6 (19%)
	Stage III, 9 (30%)	Diverticular disease, 4 (14%)

**Figure 1:**
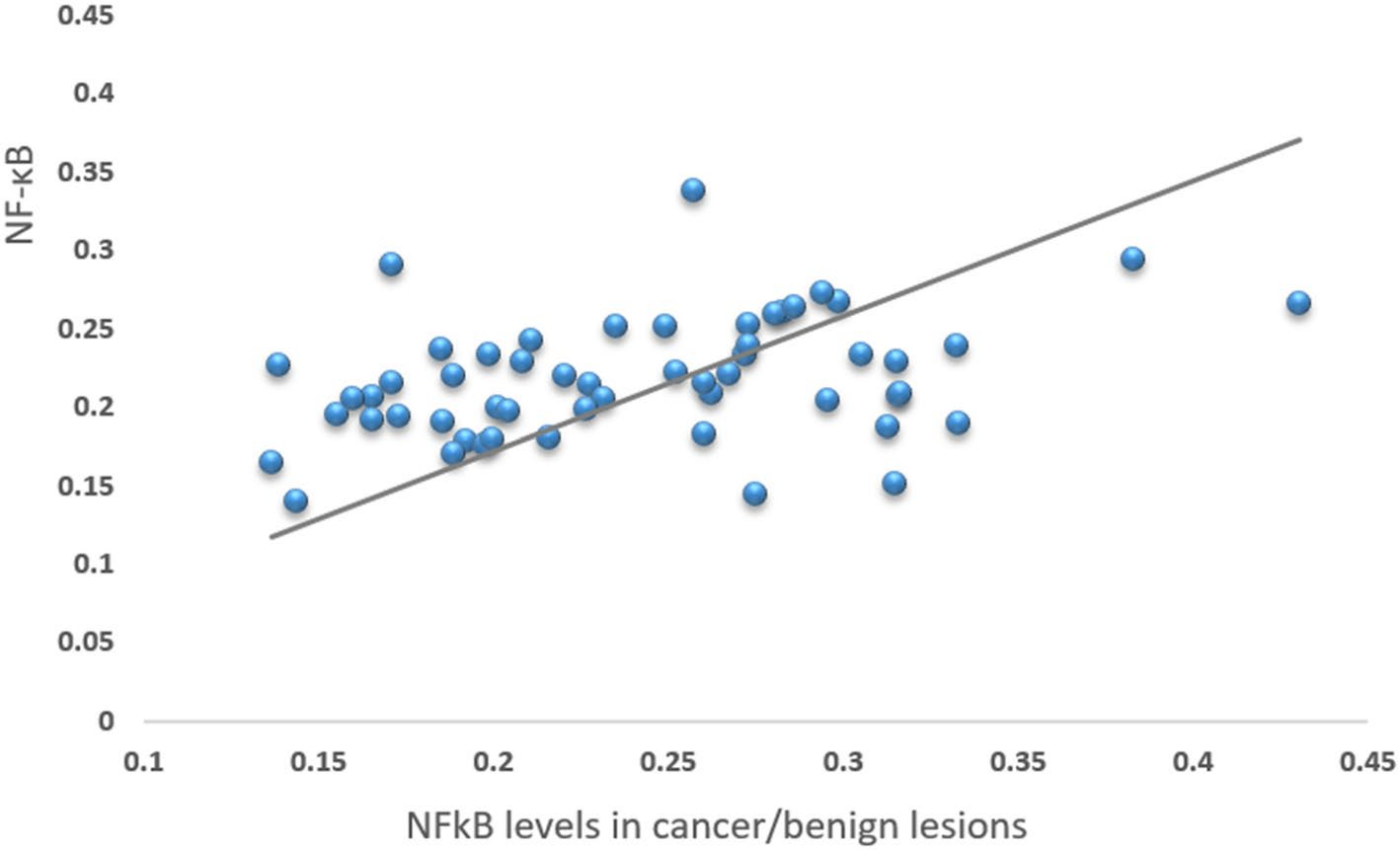
Correlation of NF-κB levels in normal vs pathological tissues. Data from all 56 patients are presented (p = 0.007).

NF-κB levels in the pathological tissue positively correlated with levels in tissue margins in all 56 patients (Fig. 1).

**Figure 2:**
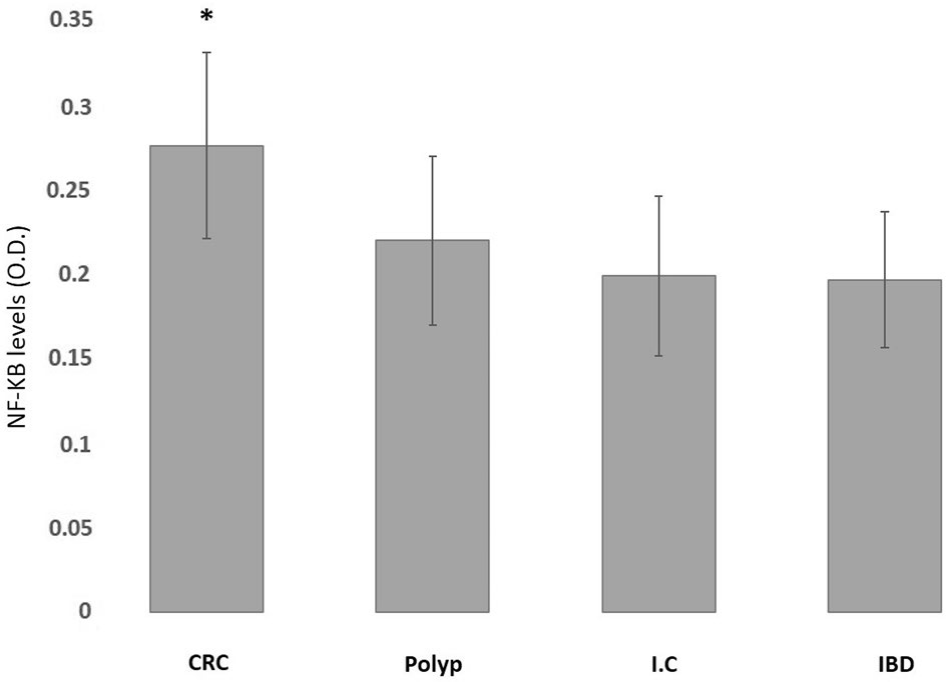
Comparison between NF-κB expression between cancerous tissues and all other pathologies (*p* < 0.005).

NF-κB expression in the cancerous tissues was significantly elevated compared to NF-κB expression in tissues of patients with adenomatous polyposis, to its expression in the epicenter of patients with all kinds of inflammatory processes and to its expression in the epicenter of the inflammation process in IBD patients (Fig. 2).

**Figure 3:**
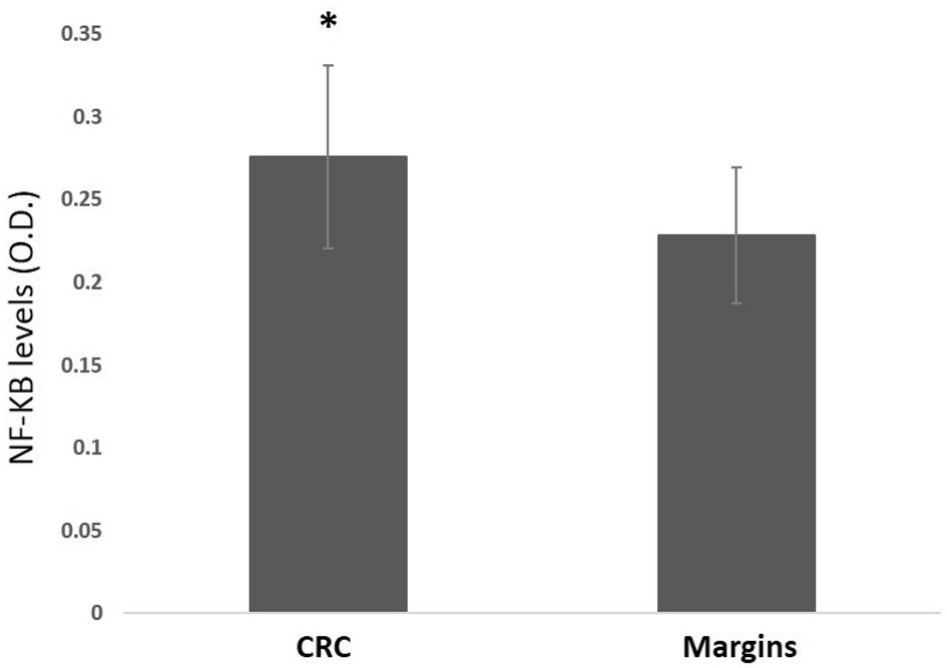
Comparison between NF-κB expression in the tumor versus the resection margins (*p* < 0. 05).

Expression of NF-Κb was higher in the cancerous tissues compared to the normal margins of resection in the same patients (Fig. 3).

Interestingly expression of NF-Κb in the inflammation epicenter of patients with IBD was similar to its expression in the resection margins of these patients. However, in 9 out of the 16 patients with IBD (56%) the resection margins had signs of inflammation in histology.

**Figure 4:**
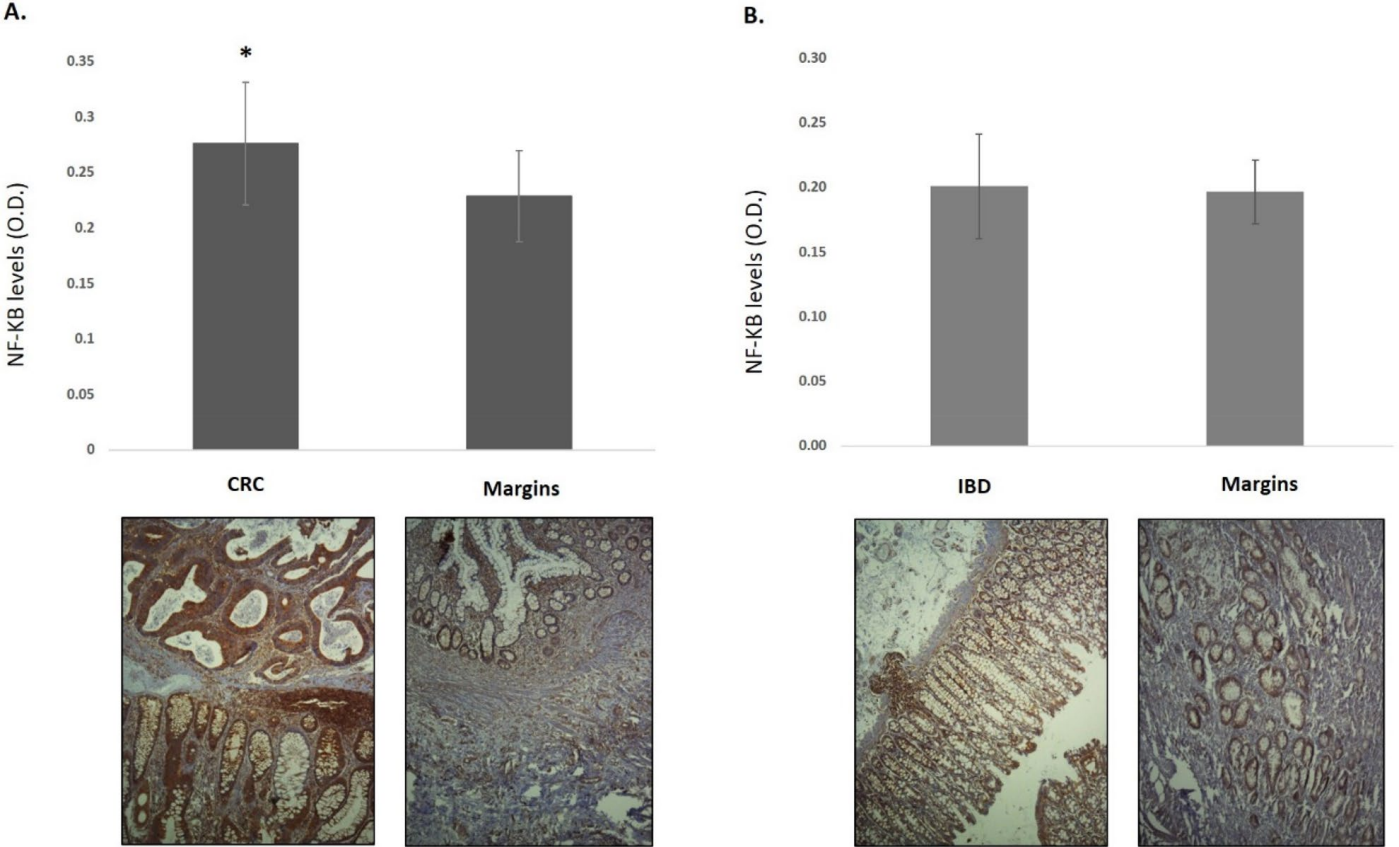
Histological images Cancer and Crohn’s disease—immunohistochemistry staining of NF-κB is seen in brown.

Histological images of cancer and Crohn’s disease are presented in Fig. 4.

A comparison of NF-κB levels between tissues of CRC patients with or without positive lymph nodes revealed elevated expression of NF-κB in the margins of CRC patients’ specimen that had positive nodes. Expression of NF-κB in the tumoral tissues was also slightly higher in the nodes’ positive group, however, it did not reach statistically significance (Table 2). NF-κB tumor expression did not correlate with the level of local tumor invasiveness (T stage).

**Table 2:** Expression of NF-κB levels in colonic tissues of CRC patients. Comparison between patients with or without positive pathological lymph nodes.

Optical density	Node positive	Node negative	p T test	p Mann w.^1^
Mean ± SD				
Tumor	0.298 ± 0.074	0.266 ± 0.043	0.147	0.354
Margins	0.249 ± 0.025	0.220 ± 0.044	0.078	0.039

^1^Mann–Whitney U test.

**Figure 5:**
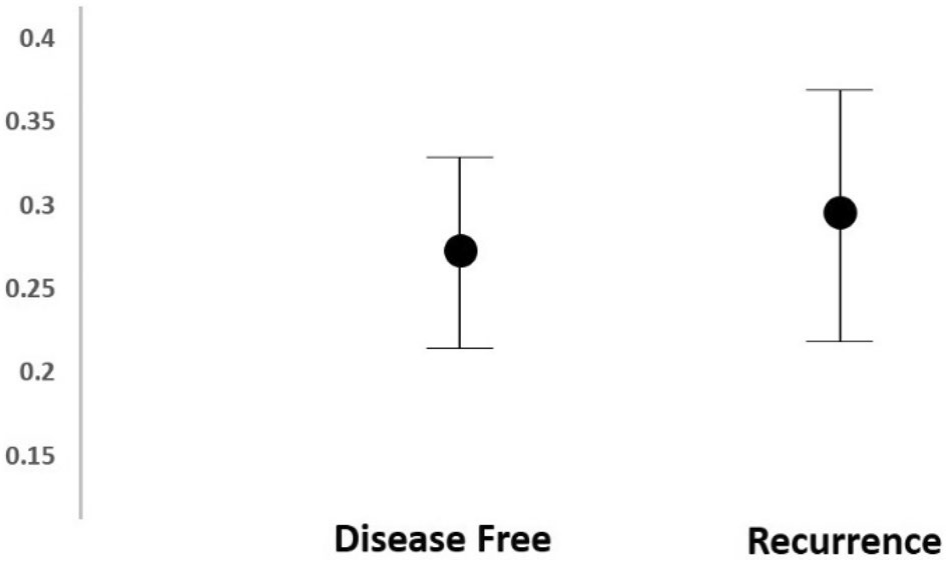
Mean ± SD of NF-kB expression. Patients with or without recurrence.

Long term follow-up revealed four patients out of the 30 cancer patients that have developed disease recurrence. The mean ± SD of NF-kB expression was compared between these 4 patients and the others (Fig. 5). A Mann–Whitney correlation test did not reveal a statistical significant difference between the two groups (p = 0.58).

**Figure 6:**
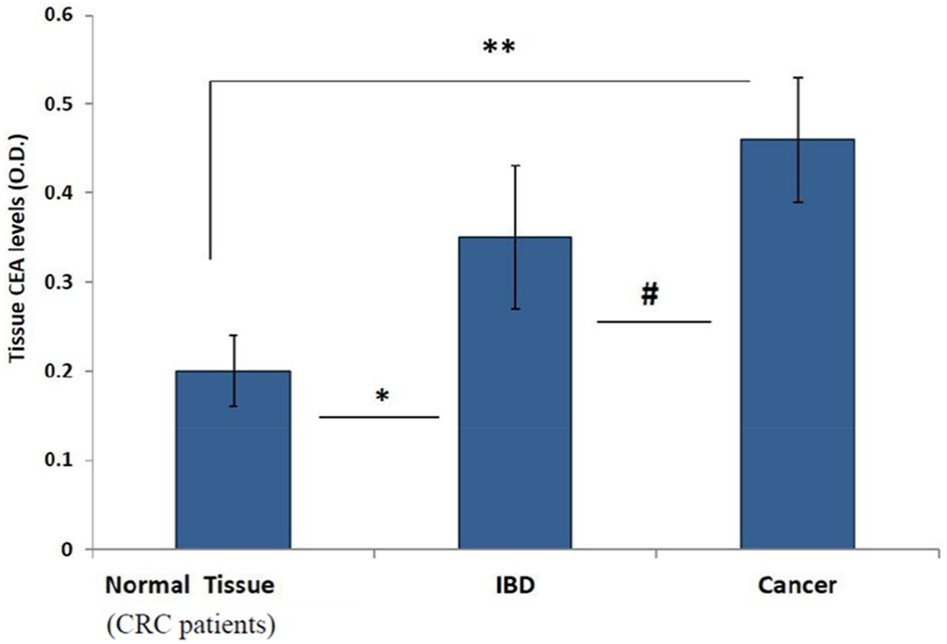
Comparison between CEA expression in tumoral tissues versus IBD and resection margins (*p* < 0. 05).

A comparison of CEA expression between cancer tissues, IBD tissues and resection margins of CRC (defined here as the control normal tissue) demonstrated high CEA expression in the cancer group, less in the IBD group and lowest in the colonic non diseased margins (Fig. 6).

A Pearson correlation test did not reveal a statistical significant correlation between CEA and NFKB expression in cancer patients (Table 3).

**Table 3:** Pearson correlation test between CEA and NFKB expression in cancer specimen.

Variables	Pearson correlations (r)	P value
CEA tumor- NFKB tumor	0.166	0.381

## Discussion

There is growing evidence linking inflammation, cancer development, and nuclear factor kappa B (NF-κB)^1^.

Preclinical studies using animal models have shown that NF-κB is involved in colorectal carcinogenesis. In mice models, the NF-κB pathway has been directly linked to intestinal inflammation and to development of colitis-associated cancer^16–18^. In mice models of CRC, NF-κB has also been linked to progression of tumor growth^17,19^.

Yet, the clinical implications of NF-κB in human inflammatory or cancer diseases of the colon have rarely been studied and evidence for its relevance to these patients is limited^20^.

To date, activating mutations of NF-κB in CRC have not been reported^3^. Nonetheless, constitutive activation of NF-κB has been observed in human CRC and associated with higher tumor stage^18,21^, treatment resistance^19,22^ and poor survival outcomes^3^. In addition, activation of the NF-κB main protein p65 in colon of metastatic CRC patients correlated with liver metastasis and poor clinical outcomes, measured as overall survival; suggesting it may have prognostic value in this disease^17,23^.

In this study, we evaluated the levels of RelA/p65 protein, which is a key in the canonical pathway of NF-κB activated by tumor necrosis factor alpha, Toll-like receptor ligands, and interleukin-1^5^. RelA/p65 is less involved in the non-canonical pathway, activated by other ligands and signals. Via the canonical pathway, different combinations of subunits with RelA/p65 protein are shown to activate this signaling pathway, regardless of the specific dimer formed^5,6^.

RelA/p65 protein was chosen based on the intent to compare protein expression between inflammatory conditions of the colon and those of cancerous conditions. In other words, since this is a basic proof of concept study we went for a gross evaluation and looked for the wide picture.

Nonetheless, here we evaluated the expression levels of RelA/p65 NF-κB protein in colonic tissues of patients undergoing surgery for various etiologies.

We have found that NF-κB levels were significantly higher in CRC cancer compared to tumor-free margins and to benign tissues, including IBD specimens. We consider this novel finding to be unpredicted since the role of the NF-κB signaling in inflammation is well-established compared to its involvement in CRC. Thus, we did not anticipate NF-κB levels to be significantly higher in CRC compared to tumor-free IBD specimens. Our findings stressed the important role of NF-κB in colorectal cancer.

Additionally, we have found that the expression of NF-κB in adenomatous polyps was similar to its expression in IBD tissues. This finding may support the hypothesis that NF-κB plays an important role early in the process of colonic dysplasia development that may lead to cancer. Interestingly, this finding correlates to other studies linking between NF-κB and colitis-associated adenoma development^24^.

Some studies tried to look for an association of NF-κB to cancer aggressiveness. Pyo et. al. investigated the correlation between phosphorylated NF-κB (pNF-κB, the activated form of the NF-κB protein) nuclear expression in pathological tissues and clinicopathological characteristics of CRC patients^20^. That study included 261 patients. They have demonstrated that pNF-κB was significantly correlated with frequent perineural invasion, lymph node metastasis and higher disease stage. The relation to disease stage was attributed to the association with positive lymph nodes but not to the tumor T stage, which was not correlated with pNF-κB levels. These findings are comparable in some aspects to our findings. Interestingly, in our study, NF-κB expression in the margins of resection was associated with positive node status of CRC patients. This may lead to the assumption that the associated processes that link NF-κB with colorectal cancer is related to the entire colonic tissue and not only to the tumor site itself. In our study, we could not show a statistical significance levels of NF-κB expression in the tumoral tissue itself between negative and positive nodes status, however, this may be related to the low number of patients we had in these groups.

Nevertheless, based on our results and others^20^, this indicates that NF-κB signaling may be involved in the dissemination processes of colonic adenocarcinoma cells. But, as its levels have not correlated with the extent of tumor involvement (T stage), we believe that NF-κB is more involved in the tumor metastatic process rather than direct invasiveness.

This data supports the hypothesis on the role of NF-κB in colorectal carcinogenesis, underlining the point that this signaling pathway may be more significant when involved in colon cancer dissemination mechanisms than in the primary colonic carcinogenic process.

In our study 4 out of 30 patients have developed recurrence. We could not show a statistical significant difference in the expression NF-κB when comparing these patients with patients that had no recurrence despite a numerical difference (Fig. 5). We believe that this may be related to the low number of patients in our series that does not allow a reliable statistical evaluation.

To further assess this hypothesis, we suggest a long-term, prospective clinical study measuring NF-κB expression levels of CRC patient colonic specimens matched with long-term follow-up data regarding metastatic disease recurrence.

Furthermore, our data revealed that NF-κB tumor levels positively correlated with expression in tissue margins (Fig. 1), which demonstrates that the activation of this signaling pathway in colons with pathological tissue is diffuse and not limited to the tumor mass. This interesting finding may suggest either a predisposition to increased NF-κB expression among patients with colonic pathologies or a remote effect of the primary lesion on the adjacent normal colonic mucosa. This also supports the concept that NF-κB may affect the metastatic potential of the tumor to its surrounding tissue more broadly than the tumor itself does.

As activation of NF-κB pathways has been previously associated with poor prognosis, it is possible that members of the NF-κB pathways could serve as prognostic markers or novel therapeutic targets for CRC. Indeed, several suggestions for anti-NF-κB therapeutics for colon cancer treatment have been examined in pre-clinical trials [reviewed in ^5,25^]. But despite the promising results, no clinical trials have been published to date. Thus, there is a lack of clinical evidence to validate this potential.

Another point regarding the association of NF-κB with poor prognosis is its role in cancer resistance to therapy. NF-κB activation has been suggested to be associated with resistance to therapy in gastrointestinal malignancies, mainly in predicting resistance to chemoradiation in esophageal cancer^26^ and to a combination of irinotecan and cetuximab in CRC^27^. Neither of these studies, however, addressed the possibility that NF-κB is a prognostic rather than a predictive clinical marker. Furthermore, the literature on the role of NF-κB in CRC progression is limited [reviewed in 5 and 25].

One of the limitation of our study is the lack of evaluation of other important biomarkers that are known to activate or be activated by NF-κB.

Two reviews on the association between the inflammation process of IBD and the development of colorectal cancer have stressed the relation between certain cytokines such as IL-6, IL- α and TNF-α to NF-κB and proposed that its mechanism of action is related to its ability to block apoptosis by positively regulating the expression of anti-apoptosis proteins^28,29^.

Our study did not aim to explore the mechanism of action of NF-κB but to demonstrate another evidence from a different angle for its pivotal role in the development of colorectal cancer.

Our study is novel in its methods (quantifying the immunochemistry staining in a relatively new method) and in its idea (comparing expression of NF-κB between cancer and IBD).

Adding a western blot and Qpcr could have further verifying the expression of NF-KB, however, the technique that we have used to quantify NF-KB is a well-established and reliable one^13,14^.

The results further support the accumulating data on the important role of NF-κB in CRC and should encourage other researchers to use this method and to further investigate the expression of NF-κB in IBD patients with and without cancer.

We have added in our study another evaluation of a biomarker, CEA. CEA is not directly related to NF-Κb and thus We did not expect to find a correlation between CEA and NF-KB. however, CEA is known to be highly associated with both CRC and IBD. Thus, the difference that we have found between CEA expression in cancer tissue compared to IBD that goes along with the difference in NF-κB expression supports the validity of our methods and findings.

## Conclusions

The results of this study add clinical evidence to the physiological involvement of NF-κB in the progression and dissemination of colon cancer. Confined with the existing literature describing the role of NF-κB in inflammation and cancer, these findings support the role of the NF-κB signaling pathway in CRC development, its potential role in tumor metastasis and its potential as a target for therapy.

Additional clinical research is needed to further validate the relevance of NF-κB tumor expression to patient prognosis in terms of recurrence and long-term survival, and clarify its role in inflammation during tumorigenesis, with or without the presence of IBD**.**

## Data Availability

The data that support the findings of this study are available on request from the corresponding author. The data are not publicly available due to privacy or ethical restrictions.
